# Supplementary material for: Risks of adverse perinatal and maternal outcomes among women with hypertensive disorders of pregnancy in southwestern Uganda
Source: PLoS One. 2020 Oct 28;15(10):e0241207. doi: 10.1371/journal.pone.0241207 (PMC7592727; doi:10.1371/journal.pone.0241207)
Supplement: S1 Questionnaire — (DOCX) [file pone.0241207.s002.docx]

# S1 Questionnaire

Date/Time of Admission

Age

Marital Status

Married

Single

Divorced

Level of Education:

None

Primary and below

O 'level

A 'level

Tertiary

__________________________________

Referral Status:

Self

Private Hospital/MD

Health Centre IV

Health Centre II or III

Other Govt Hospital

Other

Other referral status

__________________________________

Presenting Complaint

Headache

Epigastric pain

Blurring of Vision

nausea/vomiting

None

Height

__________________________________

(cm )

Weight

__________________________________

(kg )

Blood Pressure

__________________________________

HIV Status __________________________________

History of Fits prior to Admission:

Yes

No

Gravidity

__________________________________

LNMP

__________________________________

EDD

__________________________________

Gestational Age at admission

__________________________________

History of pregnancy induced hypertension in previous pregnancy

Yes

No

Date of Delivery __________________________________ Time of Delivery: _____________________________

Gestational Age at Delivery: _______________ Mode of Delivery: _______________

Birth weight _______________ Apgar Score _______________

**Fetal complication {tick appropriately}**

YES

NO

Fresh still birth

Macerated still birth

Admission to Neonatal unit

Did any of the following fetal events occur during

Neonatal death < 24hrs following delivery

hospitalization?

Neonatal death prior to discharge

No events (6)

Antihypertensive given: Yes/No

Dexamethasone given: Yes/No

magnesium sulfate

Received loading dose only

Received both loading and complete maintenance dose

Received both loading and incomplete maintenance

dose

Did not receive

Did the participant have any of the following events

Uterine rupture

during her hospitalization?

Uterine dehiscence

Hysterectomy

Laparotomy

Blood Transfusion

Re-operation

ICU Admission

Surgical Injury (Bowel/Bladder/Ovary/Fallopian

Tube)

Need for blood transfusion (blood unavailable)

Maternal death < 24hrs after delivery

Hospitalization >1 days for a vaginal delivery

Hospitalization >3 days for caesarean delivery

Maternal death prior to discharge

postpartum hemorrhage

coagulopathy (DIC)

eclampsia

No events

**Maternal complication {tick appropriately}**

YES

NO

Death

ICU admission

Hemodialysis

Stroke

Hellp

Acute Kidney Injury

pulmonary edema or respiratory

failure
